# Lysophosphatidylcholine acyltransferase 1 promotes head and neck squamous cell carcinoma progression by enhancing COX17-dependent oxidative phosphorylation

**DOI:** 10.1038/s41420-026-02994-3

**Published:** 2026-03-06

**Authors:** Yuanyang Zhao, Yun Li, Yanshi Li, Zhihai Wang, Chuan Liu, Lin Chen, Min Wang, Mengna Wang, Zhaobo Cheng, Guohua Hu, Min Pan

**Affiliations:** 1 Department of Otorhinolaryngology, The First Affiliated Hospital of Chongqing Medical University, Chongqing, P. R. China; 2 The First Clinical College, Chongqing Medical University, Chongqing, P. R. China; 3 Center for Lipid Research, The Second Affiliated Hospital of Chongqing Medical University, Chongqing, P. R. China

**Keywords:** Oral cancer, Cancer

## Abstract

Metabolic dysregulation is increasingly recognized as a driver of tumor progression, yet its specific role in head and neck squamous cell carcinoma (HNSCC) remains poorly characterized. This study integrated untargeted metabolomics of HNSCC patient tissues with multi-omics validation to identify key metabolic alterations. We discovered a significant accumulation of sn-2 saturated fatty acyl-phosphatidylcholines, implicating disrupted phospholipid remodeling in HNSCC pathogenesis. Analysis of The Cancer Genome Atlas and Human Protein Atlas databases revealed consistent upregulation of lysophosphatidylcholine acyltransferase 1 (LPCAT1) in HNSCC. This finding was further validated at mRNA, protein, and tissue levels by quantitative PCR, western blotting, and immunohistochemistry, respectively. Functional assays demonstrated that LPCAT1 knockdown suppressed tumor cell proliferation, migration, and invasion while increasing cell death in vitro, and inhibited tumor growth in nude mouse xenograft models. Mechanistically, LPCAT1 depletion impaired mitochondrial oxidative phosphorylation by reducing Cytochrome c oxidase activity, thereby decreasing ATP production. Our data further demonstrate that LPCAT1 regulates the expression of COX17, suggesting that the promotion of Cytochrome c oxidase activity and tumor bioenergetics by LPCAT1 is mediated through COX17. Thus, LPCAT1 drives HNSCC progression via a COX17-dependent metabolic reprogramming pathway. Targeting LPCAT1 represents a promising therapeutic strategy, while tissue-saturated fatty acyl-phosphatidylcholines may serve as early diagnostic biomarkers for HNSCC.

## Introduction

Head and neck squamous cell carcinoma (HNSCC), the sixth most common global malignancy, faces rising incidence with 1.08 million annual cases projected by 2030 [1]. Despite improved early-stage survival [2], 60% of patients present with locally advanced disease (stage III–IV B), and 5-year survival remains stagnant at 50% [3, 4]. The pathogenesis, progression, and metastasis of HNSCC involve multiple mechanisms, such as TP53 variants [5], activation of the EGFR signaling pathway [6], and epithelial-mesenchymal transition [7], yet remain incompletely understood. These challenges highlight the urgent need for novel therapeutic targets and reliable biomarkers.

It is now well-established that cancers undergo extensive metabolic reprogramming to fuel their uncontrolled growth and survival [8, 9]. This adaptation extends beyond aerobic glycolysis [10] to encompass profound alterations in lipid, amino acid, and nucleotide metabolism [9, 11]. These changes not only meet the bioenergetic and biosynthetic demands but also generate signaling molecules that actively promote oncogenic processes [12].

Importantly, tumor-associated metabolites serve a dual role. On one hand, their specific alteration patterns can serve as valuable biomarkers for early detection [13], prognosis [14], or monitoring treatment response [15]. On the other hand, many metabolites are not merely passive end products; they possess bioactivity [16] and can directly influence critical cellular functions, including signal transduction, epigenetic regulation, and cell fate decisions [17, 18]. The precise roles of dysregulated metabolites in HNSCC progression, however, are largely unexplored.

The aberrant levels of metabolites are primarily driven by the dysregulation of metabolic enzymes. These enzymes act as key executors of metabolic reprogramming [19] and represent promising therapeutic targets [20, 21], as their inhibition can directly disrupt oncogenic metabolic fluxes crucial for tumor survival and growth. Therefore, identifying the key dysregulated metabolites and their corresponding regulatory enzymes in HNSCC is of paramount importance.

Through an unbiased metabolomics approach, our study identified aberrant alterations in the phosphatidylcholine (PC) profile and abundance in HNSCC. This dysregulation particularly involved specific PC species, including PC (16:0/16:0) (1,2-dipalmitoyl-sn-glycero-3-phosphocholine, DPPC) and PC (18:1/16:0), synthesized by the enzyme lysophosphatidylcholine acyltransferase 1 (LPCAT1) [22]. Analysis of the TCGA database confirmed that LPCAT1 is significantly upregulated in HNSCC tumor tissues. We demonstrated that LPCAT1 is essential for HNSCC cell proliferation, migration, and tumor growth in vivo. Mechanistically, we uncovered a novel function of LPCAT1 in enhancing mitochondrial oxidative phosphorylation (OXPHOS) by upregulating the expression of COX17, thereby boosting Cytochrome c oxidase activity. Our work identifies PC metabolism as a potential biomarker for the early diagnosis of HNSCC, and targeting LPCAT1 holds promise as a potential therapeutic strategy for HNSCC.

## Results

### PC accumulates significantly in the tumor tissues of HNSCC patients

**Fig. 1:**
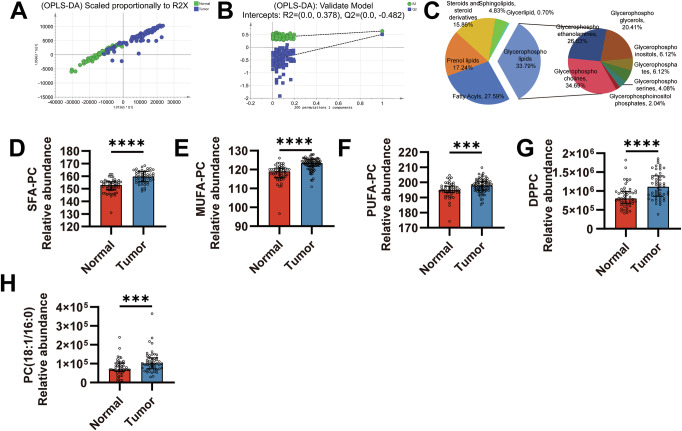
Metabolomic analysis reveals alterations in phosphatidylcholine composition in tumor tissues of HNSCC patients. **A** OPLS-DA score plots. **B** OPLS-DA permutation test (Q2 = −0.482). **C** Proportion of lipid metabolites. The relative abundance of SFA-PC (**D**), MUFA-PC (**E**), and PUFA-PC (**F**) in HNSCC and normal tissues. The relative abundance of DPPC (**G**) and PC (18:1/16:0) (**H**) in HNSCC and normal tissues. For tumor tissues, *n* = 81; for normal tissues, *n* = 50. Statistical significance determined by Mann–Whitney test (**D**–**H**). ***P < 0.001; ****P < 0.0001.

To comprehensively characterize metabolic alterations in HNSCC, we conducted untargeted metabolomic profiling on 81 HNSCC and 50 normal tissues from HNSCC patients. OPLS-DA revealed distinct metabolic profiles between the groups (Fig. 1A; R2X = 0.612, R2Y = 0.645, Q2 = 0.515), with model robustness confirmed by permutation testing (Fig. 1B; Q2 = −0.482). This indicates significant global metabolic reprogramming in HNSCC tissues. Identification of differential metabolites (VIP > 1, *P* < 0.05) highlighted phospholipids as the most significantly altered lipid class, accounting for 33.79% of total dysregulated lipids. Within this class, PCs constituted the predominant dysregulated subclass, representing 34.69% of differential phospholipids (Fig. 1C). This specific PC accumulation indicates perturbed PC metabolism in HNSCC.

Variations in acyl chain saturation at the sn-2 position of PC significantly modulate disease pathogenesis, establishing acyl remodeling at this site as central to its metabolic regulation [23–25]. Based on these findings, this study focused on the in-depth characterization of PC subspecies. Compared with normal tissues, PC subspecies containing saturated fatty acids (SFAs), monounsaturated fatty acids (MUFAs), and polyunsaturated fatty acids (PUFAs) at the sn-2 position exhibited abnormal accumulation (Fig. 1D–F). Notably, the increases in SFA-PC and MUFA-PC levels were particularly pronounced. Furthermore, marked enrichment of two specific PC subspecies in tumor tissues, DPPC and PC (18:1/16:0), was observed (Fig. 1G, H). These data indicate that the metabolic profile in HNSCC tumor tissues is altered compared to normal tissues, characterized primarily by the accumulation of PC, suggesting that PC may serve as a biomarker for early detection of HNSCC, and that the corresponding PC metabolic pathways may be dysregulated in HNSCC.

### LPCAT1 expression was increased in HNSCC tissues

**Fig. 2:**
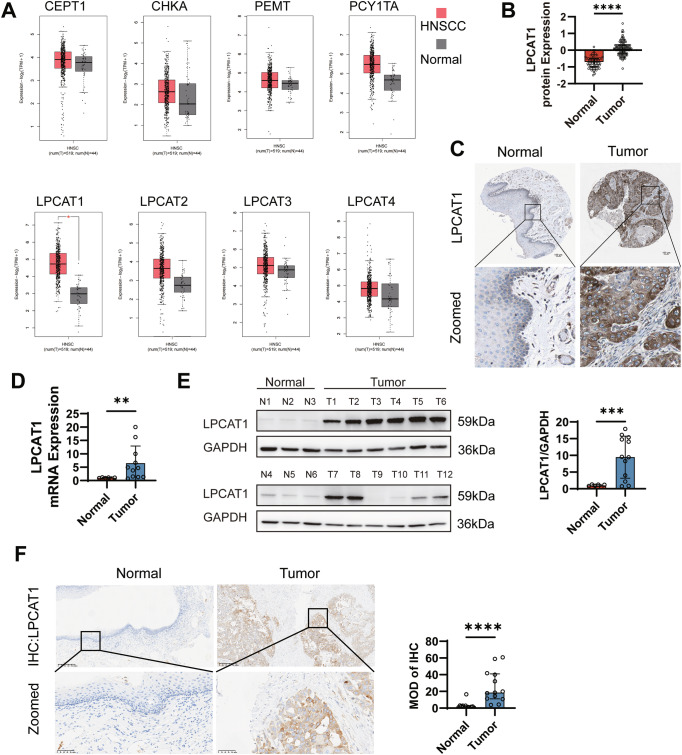
LPCAT1 expression was increased in HNSCC tissues. **A** Transcriptomic analysis of the TCGA database shows the mRNA expression levels of phosphatidylcholine-metabolizing enzymes, including LPCAT1, CEPT1, CHKA, PEMT, PCYT1A, LPCAT2, LPCAT3, and LPCAT4, in HNSCC (*n* = 519) and normal tissues (*n* = 44). **B** LPCAT1 protein expression in HNSCC (*n* = 109) and normal tissues (*n* = 70) from the Human Protein Atlas database. **C** Representative immunohistochemical staining of LPCAT1 protein in normal oral mucosa and HNSCC from the Human Protein Atlas database. Scale bar, 100 μm in the upper image, 20 μm in the lower image. LPCAT1 mRNA (**D**) and protein (**E**) in normal tissues (*n* = 6) and tumor tissues (*n* = 12) were measured by qPCR and western blotting, respectively. **F** Representative immunohistochemical staining of normal tissues (*n* = 13) and tumor tissues (*n* = 13) with LPCAT1 antibody. Scale bar, 200 μm in the upper image, 50 μm in the lower image. Statistical significance determined by Mann–Whitney test (**B**, **F**) or Student’s *t* test (**A**) or Student’s *t* test with Welch’s correction (**D**, **E**). *P < 0.05; **P < 0.01; ***P < 0.001; ****P < 0.0001.

Metabolic alterations were primarily due to the irregular expression of metabolic enzymes [26]. We analyzed the major PC synthesis and remodeling pathways (Kennedy pathway, PEMT pathway, Lands cycle) in HNSCC using the TCGA-HNSC cohort. As shown in Fig. 2A, only LPCAT1 expression was significantly elevated in the tumor, which was consistent with the abnormal accumulation of DPPC and PC (18:1/16:0) [22]. However, the expression levels of other key PC metabolism enzymes, including CEPT1, CHKA, PEMT, PCYT1A, LPCAT2, LPCAT3, and LPCAT4, showed no significant difference. Accordingly, at the protein level, data from the Human Protein Atlas show that LPCAT1 protein expression is significantly higher in HNSCC tumor tissues than in normal tissues (Fig. 2B, C). To validate whether LPCAT1 expression is abnormal in the HNSCC tumor tissues exhibiting significant metabolic profile differences, we performed verification using qPCR, western blotting, and immunohistochemistry. As shown in Fig. 2D–F, the mRNA and protein expression of LPCAT1 in 12 tumor tissues was significantly higher than that in 6 normal tissues, suggesting that aberrant expression of the key PC metabolism enzyme LPCAT1 plays an important role in HNSCC.

### LPCAT1 knockdown suppresses HNSCC cell proliferation, migration, and invasion in vitro

**Fig. 3:**
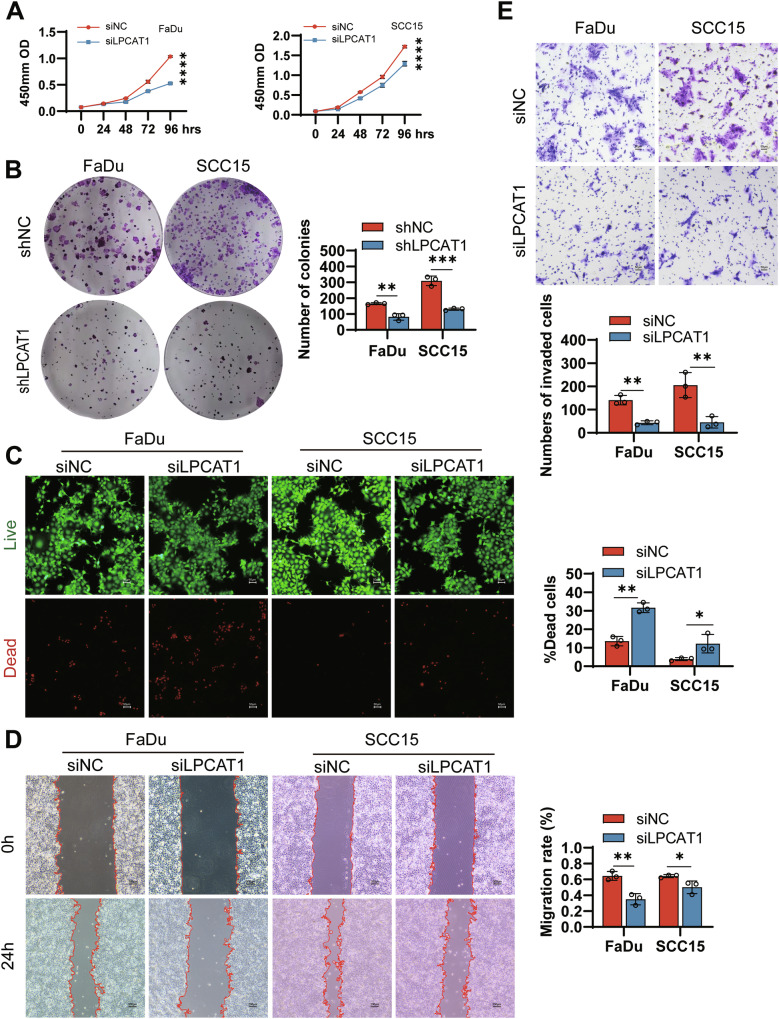
LPCAT1 knockdown suppresses HNSCC cell proliferation, migration, and invasion in vitro. **A** CCK-8 assay was used to detect the proliferation of FaDu and SCC15 cells with or without LPCAT1 knockdown. **B** Cell growth was measured by colony formation assays. **C** Live/Dead staining for cell death measurement. Representative images were shown. Green represents live cells, and red represents dead cells. The percentage of dead cells was calculated. Scale bar, 50 μm. **D** Wound-healing assays were used to detect cell migration. Scale bar, 200 μm. **E** Transwell invasion assays were used to evaluate cell invasive potential. Scale bar, 50 μm, *n* = 3 for (**A**–**E**). Statistical significance determined by Student’s *t* test (**B**–**E**) or two-way ANOVA (**A**). *P < 0.05; **P < 0.01; ***P < 0.001; ****P < 0.0001.

To investigate the functional role of LPCAT1 in the development and progression of HNSCC, we conducted in vitro experiments in FaDu and SCC15 cell lines. Knockdown of LPCAT1 significantly inhibited the proliferation (Fig. 3A) and colony formation (Fig. 3B) of FaDu and SCC15 cells. And LPCAT1 knockdown also promoted cell death in both cell lines (Fig. 3C). To further investigate the role of LPCAT1 in cell migration and invasion, we performed scratch assays and transwell assays. As shown in Fig. 3D, E, after LPCAT1 knockdown, wound closure was delayed, and cell invasion through the matrigel was reduced, indicating that LPCAT1 knockdown impairs the migration and invasion of FaDu and SCC15 cells. Taken together, LPCAT1 plays a positive promoting role in the progression of HNSCC.

### LPCAT1 knockdown suppresses HNSCC tumor growth in vivo

**Fig. 4:**
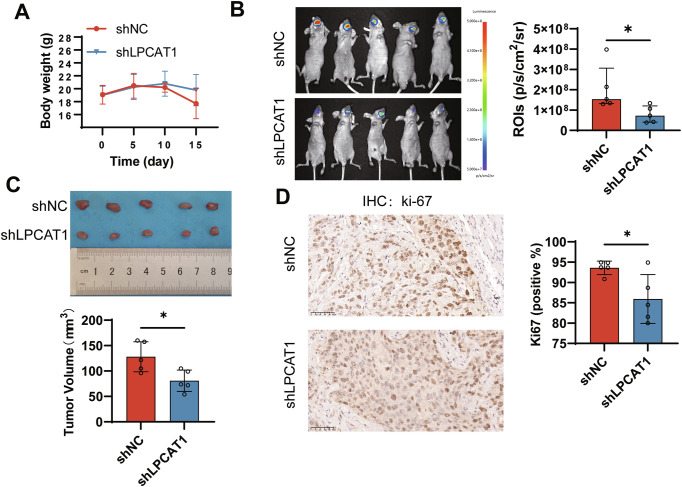
LPCAT1 knockdown suppresses tumor growth in vivo. **A** Body weight was monitored every 5 days for 15 days after tumor implantation. **B** In vivo fluorescence imaging of FaDu-shLPCAT1 and FaDu-shNC xenografts at endpoint and quantification of fluorescence intensity from endpoint imaging. **C** Tumor volume was measured at the endpoint. Statistical analysis of tumor volume differences between the LPCAT1 knockdown and control groups. **D** Representative immunohistochemical staining of tumor tissues from FaDu-shLPCAT1 and FaDu-shNC xenografts with Ki-67 antibody. Scale bar, 50 μm. The percentages of Ki-67-positive cells were calculated. Statistical significance determined by Student’s *t* test (**C**), Student’s *t* test with Welch’s correction (**D**), Mann–Whitney test (**B**). *P < 0.05.

To further elucidate the role of LPCAT1 in vivo, we established an orthotopic tongue xenograft model. FaDu cells with stable LPCAT1 knockdown (shLPCAT1) or control cells (shNC) were established and then implanted into nude mice, respectively. Longitudinal monitoring revealed that some mice exhibited >20% body weight loss by day 15 (Fig. 4A), necessitating experimental termination per ethical guidelines. Subsequent bioluminescent imaging showed that the tumor fluorescence intensity in the LPCAT1-knockdown group was significantly lower than that in the control group (Fig. 4B). Further, after tumor collection, we observed that the tumor volume in the LPCAT1-knockdown group was significantly smaller than that in the control group, indicating that LPCAT1 knockdown suppresses tumor growth (Fig. 4C). To further evaluate tumor cell proliferation, we performed immunohistochemical staining for Ki-67 on the tumor tissues from both groups. We observed that the number of Ki-67-positive cells in the LPCAT1 knockdown group was significantly lower than that in the control group (Fig. 4D), indicating that LPCAT1 knockdown suppresses tumor cell proliferation. Collectively, these data establish LPCAT1 as a critical regulator of HNSCC tumor progression both in vitro and in vivo.

### LPCAT1 promotes oxidative phosphorylation in FaDu cell

**Fig. 5:**
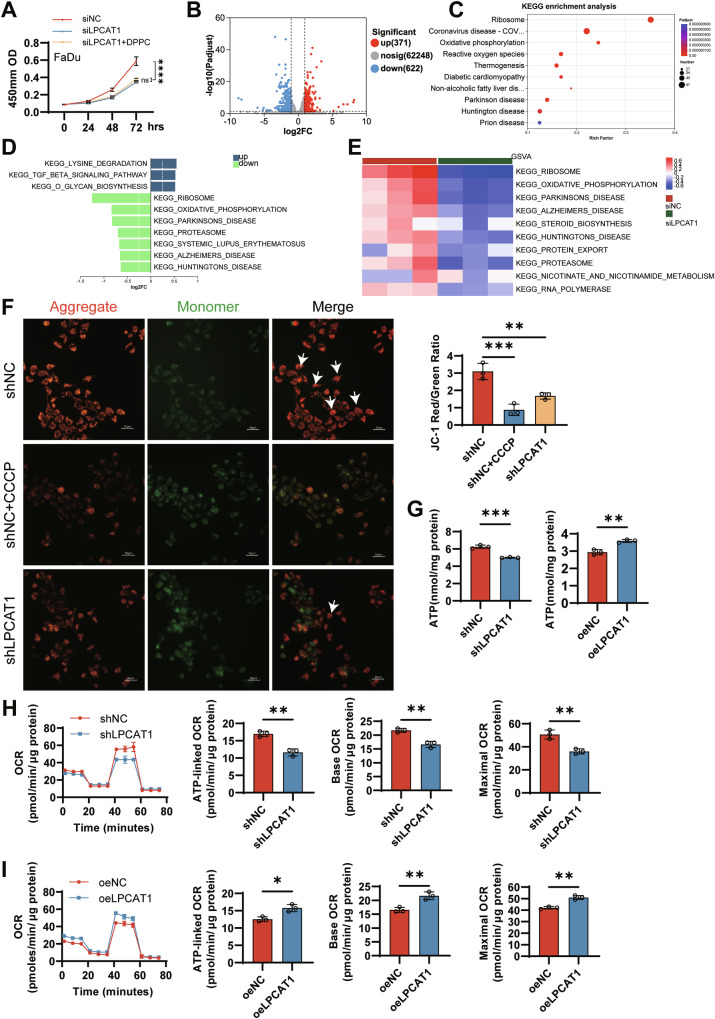
LPCAT1 promotes mitochondrial oxidative phosphorylation in FaDu cells. **A** CCK-8 assay was used to monitor cell proliferation. **B** Volcano plot of differentially expressed genes (DEGs) in LPCAT1-knockdown (FaDu-siLPCAT1) and control (FaDu-siNC) cells, showing 622 downregulated and 371 upregulated genes (fold change ≥2, *P*adjust <0.05). **C** KEGG pathway enrichment analysis of differentially expressed genes (*P*adjust <0.01). **D** Differential pathway activity between LPCAT1-knockdown and control groups (*P*adjust <0.05). **E** Heatmaps displaying GSVA enrichment scores per sample for each module. **F** The mitochondrial membrane potential was detected by JC-1 staining. Images were acquired using fluorescence microscopy. Scale bar, 50 μm. **G** ATP production was measured by quantification assays following LPCAT1 knockdown or overexpression. Mitochondrial respiration was measured by Seahorse XFp analysis following LPCAT1 knockdown (**H**) or overexpression (**I**). Basal OCR, ATP-linked OCR, and maximal OCR were calculated. *n* = 3 for (**A**–**I**). Statistical significance determined by two-way ANOVA test (**A**), or one-way ANOVA with Dunnett’s multiple comparison test (**F**), or Student’s *t* test (**G**–**I**). *P < 0.05, **P < 0.01, ***P < 0.001.

The DPPC restored EGFR signaling and cell proliferation in LPCAT1-depleted glioblastoma cells [27] and mediated the activation of ERK1/2-CREB signaling in hepatocellular carcinoma [28]. To elucidate the role and underlying mechanism of LPCAT1 and DPPC in HNSCC, we supplemented DPPC in FaDu cells with LPCAT1 knockdown. Surprisingly, DPPC supplementation had little or no rescuing effect on FaDu cell proliferation, which contrasts with the pronounced rescue effect observed in glioblastoma cells (Fig. 5A). This divergence suggests that the oncogenic function of LPCAT1 in HNSCC may be independent of its production of this specific PC species or that DPPC alone is insufficient. To elucidate the molecular mechanisms by which LPCAT1 prompts tumor progression, we performed transcriptome sequencing in LPCAT1-knockdown and control cells. We identified 993 differentially expressed genes, comprising 622 downregulated and 371 upregulated genes (Fig. 5B). To understand the specific functional roles of the differentially expressed genes, KEGG enrichment analysis was performed. As shown in Fig. 5C, the oxidative phosphorylation pathway was significantly affected. Moreover, GSVA revealed that OXPHOS pathway activity was decreased (Fig. 5D, E), suggesting a link between LPCAT1 and mitochondrial energy metabolism.

Mitochondrial membrane potential serves as a key physiologic indicator for assessing cellular ATP production capacity through OXPHOS [29, 30]. To determine the impact of LPCAT1 knockdown on mitochondrial energy metabolism, we detected changes in mitochondrial membrane potential using the JC-1 fluorescent probe. As shown in Fig. 5F, compared with the control group, the ratio of JC-1 red to green fluorescence intensity in LPCAT1 knockdown cells decreased by approximately 1.8-fold. Moreover, LPCAT1 knockdown reduced ATP production, while LPCAT1 overexpression increased ATP production, suggesting that LPCAT1 increased mitochondrial energy metabolism in HNSCC cells (Fig. 5G). Further, we measured cellular OCR to assess mitochondrial function and energy metabolism status. LPCAT1 knockdown significantly reduced basal OCR, ATP-linked OCR, and maximal OCR (Fig. 5H), while LPCAT1 overexpression increased these parameters (Fig. 5I). Hyperactivated energy metabolism promoting tumor progression in HNSCC has been previously reported [31, 32]. Therefore, we conclude that LPCAT1 promotes tumor development by upregulating OXPHOS activity and energy metabolism in HNSCC cells.

### LPCAT1 enhances the activity of CcO by upregulating COX17 expression, thereby promoting OXPHOS in HNSCC

**Fig. 6:**
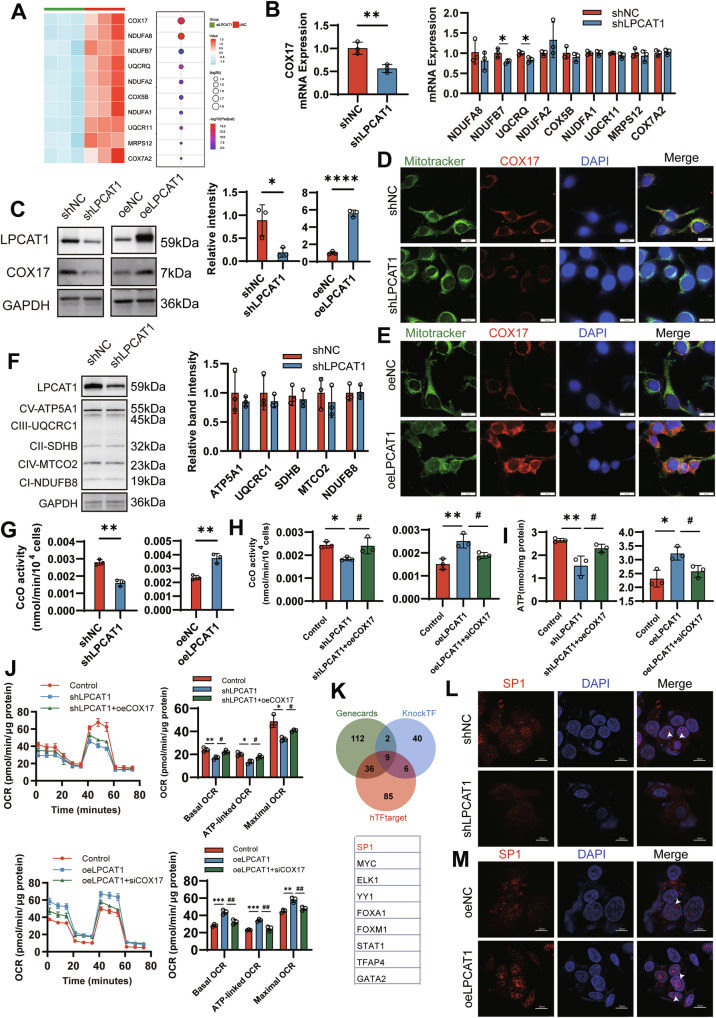
LPCAT1 enhances cytochrome c oxidase activity by upregulating COX17 expression in HNSCC. **A** Transcriptomic analysis highlighting COX17 as a top dysregulated gene within the oxidative phosphorylation pathway in LPCAT1-knockdown cells. **B** qPCR validation of top 10 genes mRNA levels. **C** COX17 protein was measured by western blotting after LPCAT1 knockdown or overexpression. Representative immunofluorescence staining of LPCAT1 knockdown (**D**) or overexpression (**E**) cells with mitotracker and COX17 antibody. Images were acquired using confocal microscopy. Scale bar, 20 μm. **F** Western blotting analysis of electron transport chain complex core subunits (I–V). **G** Microplate assay measurement of CcO activity after LPCAT1 knockdown or overexpression. Overexpress COX17 in LPCAT1-knockdown cells or silence COX17 in LPCAT1-overexpression cells. CcO activity (**H**) and ATP production (**I**) were detected, and a mitochondrial stress test (**J**) was performed. **K** The potential transcription factors of COX17 were predicted by Genecard, KnockTF, and hTFtarget. Representative immunofluorescence staining of LPCAT1 knockdown (**L**) or overexpression (**M**) cells with SP1 antibody. Scale, 20 μm. SP1 in the nucleus is indicated by white arrows. *n* = 3 for (**B**–**J**, **L**, **M**). Statistical significance determined by Student’s *t* test (**B**, **C**, **F**, **G**), or one-way ANOVA with Tukey’s multiple comparison test (**H**–**J**). *P < 0.05; **P < 0.01, ***P < 0.001, ^#^*P* < 0.05, ^##^*P*<0.01.

To identify the mechanism mediating LPCAT1-induced enhancement of oxidative phosphorylation, we analyzed the genes related to the oxidative phosphorylation pathway from the transcriptome sequencing data. As shown in Fig. 6A, COX17 is the most significantly differentially expressed gene. qPCR analysis confirmed that COX17 mRNA expression was significantly lower in LPCAT1 knockdown cells compared to control cells (Fig. 6B). We further examined COX17 protein levels in cells and COX17 levels in mitochondria by immunoblotting and immunofluorescence, respectively. Consistent with the mRNA expression, LPCAT1 knockdown reduced COX17 protein expression, while LPCAT1 overexpression increased mitochondrial COX17 protein levels (Fig. 6C–E and Figs. S1 and S2).

Given the critical role of COX17 in mitochondrial respiration, particularly in the activity of CcO [33], we investigated how LPCAT1 affects OXPHOS through COX17. To determine whether LPCAT1 affects ETC complexes, we analyzed the core subunits of all respiratory complexes (I–V). We did not observe any changes in the expression of the five complexes (Fig. 6F). Interestingly, isolated mitochondrial assays showed that LPCAT1 knockdown reduced the activity of CcO (Complex IV), while its overexpression enhanced the enzyme’s activity (Fig. 6G). To demonstrate that LPCAT1 enhances CcO activity through COX17, we performed a rescue experiment. Overexpression of COX17 in LPCAT1-knockdown cells restored CcO activity, while knockdown of COX17 in LPCAT1-overexpressing cells reduced the LPCAT1-induced increase in CcO activity (Fig. 6H). Accordingly, overexpression of COX17 in LPCAT1-knockdown cells restored ATP production and increased mitochondrial basal OCR, ATP-linked OCR, and maximal OCR; conversely, COX17 knockdown in LPCAT1-overexpressing cells reduced LPCAT1-induced ATP production and decreased mitochondrial basal OCR, ATP-linked OCR, and maximal OCR (Fig. 6I, J). These results indicate that COX17 expression is a critical mediator of LPCAT1-promoted oxidative phosphorylation. Previous studies have shown that SP1 is involved in the regulation of OXPHOS-related genes [34]. Specifically, the promoter activity of the COX17 gene in mice depends on SP1 binding [35]. Through predictions from the hTFtarget, GeneCards, and KnockTF databases, we found that SP1 also serves as a transcription factor for human COX17 (Fig. 6K). Since LPCAT1 has been reported to promote SP1 nuclear translocation in esophageal squamous cell carcinoma [36], we hypothesized that LPCAT1 might similarly affect nuclear SP1 levels in HNSCC cells. As confirmed in Fig. 6L, M, LPCAT1 knockdown reduced nuclear SP1 expression, whereas LPCAT1 overexpression increased nuclear SP1 levels. These findings suggest that LPCAT1 may regulate mitochondrial energy metabolism by promoting SP1 nuclear translocation and subsequent SP1-mediated transcriptional upregulation of COX17. Taken together, COX17 is a key mediator through which LPCAT1 maintains CcO activity and OXPHOS.

## Discussion

This study innovatively uncovers the role and underlying mechanisms of LPCAT1, a key enzyme in PC metabolism in HNSCC, and the aberrant accumulation of PC in this malignancy. We provide the first evidence that LPCAT1 enhances mitochondrial oxidative phosphorylation by upregulating COX17-dependent CcO activity, thereby driving HNSCC progression. Overall, PC has the potential to serve as a biomarker for early warning and diagnosis of HNSCC, and targeting LPCAT1 represents a promising therapeutic strategy for HNSCC.

Emerging evidence indicates that abnormal levels of metabolites and their associated metabolic enzymes may both regulate tumor cell proliferation, differentiation, immune evasion, and metastasis [37, 38]. LPCAT1 has emerged as a critical metabolic modulator that fuels tumor progression through distinct pathways across malignancies. Previous studies have reported that DPPC, a major metabolic product of LPCAT1 [39], modulates cellular membrane phospholipid composition, thereby activating the ERK1/2-CREB signaling pathway to promote hepatocellular carcinoma progression [28]. Similarly, this lipid alters the biophysical properties of glioma cell membranes, stabilizes and enhances oncogenic EGFR signaling, and drives tumor progression [27]. In this study, we also observed a significant accumulation of DPPC in HNSCC. To investigate the potential role of this accumulation in promoting HNSCC, preliminary functional assays were performed. Our data showed that DPPC had no effect on FaDu cell proliferation after LPCAT1 knockdown. This suggests that DPPC may influence other aspects of HNSCC biology rather than proliferation. Notably, DPPC rescued resistance to PUFA toxicity [40]. LPCAT1 enhanced membrane phospholipid saturation to increase cancer cell resistance to ferroptosis [41]. Thus, while specific PC subspecies may play distinct roles in oncogenesis, our data indicate that in HNSCC, LPCAT1 operates through alternative mechanisms.

In fact, aside from the effects of its metabolites, LPCAT1 can also contribute to tumor progression via other metabolic functions. For instance, in esophageal squamous cell carcinoma, LPCAT1 drives progression by reprogramming cholesterol metabolism [36]. Our study reveals that LPCAT1 promotes tumorigenesis via a distinct and critical mechanism, the enhancement of OXPHOS in HNSCC. Activation of OXPHOS is critical for meeting the heightened energy demands of aggressive tumors, serving as a central mechanism for cancer cell progression [42, 43]. For example, in RB1-deficient breast cancer, upregulated OXPHOS directly supports anabolic metabolism and cell proliferation while providing an energetic basis for metastatic dissemination [44]. Notably, OXPHOS represents an important metabolic signature in cancer stem cells across multiple tumor types, with strong associations to tumor recurrence, metastasis, and drug resistance [45]. In HNSCC, elevated OXPHOS activity correlates with poor clinical outcomes and therapeutic resistance, particularly in tumor stem cells exhibiting metabolic phenotypes deviating from the classical Warburg effect [46–48]. Our study establishes LPCAT1 as a key enhancer of mitochondrial OXPHOS in HNSCC. This discovery aligns with LPCAT1’s recently reported role in elevating energy status in triple-negative breast cancer. LPCAT1 enhances ATP levels and ATPase activity, promoting nuclear translocation of the BAF complex containing DPF2 subunit, which upregulates TGFBR2 transcription and subsequently activates the TGFβ signaling pathway to drive proliferation and metastasis [49]. Collectively, these observations position LPCAT1 as a key driver of HNSCC progression through its ability to potentiate OXPHOS.

The electron transport chain (ETC) is well-established as essential for OXPHOS. Mechanistically, we found that LPCAT1 critically regulates the expression of COX17. Knockdown of LPCAT1 significantly reduced COX17 expression, leading to impaired Complex IV activity and consequent OXPHOS dysfunction. This mitochondrial impairment provides a direct explanation for the apoptosis induced by LPCAT1 knockdown reported in previous studies [36, 50]. As ETC dysfunction, collapse of mitochondrial membrane potential, and ATP depletion are potent triggers of the intrinsic apoptotic pathway [51–53].

Notably, beyond compromising mitochondrial energy production, the loss of COX17 is known to disrupt cellular copper homeostasis [54], blocking CcO activity [33]. Critically, copper homeostasis induced cuproptosis by forcing oligomerization of lipoylated proteins [55]. Given our observation of reduced COX17 expression and mitochondrial dysfunction upon LPCAT1 depletion, we speculate that in HNSCC, loss of LPCAT1 may not only trigger apoptosis via OXPHOS impairment but could also potentially induce cuproptosis through copper dyshomeostasis. This interplay warrants further investigation.

Furthermore, ETC is located in the inner mitochondrial membrane, and its function is highly dependent on the phospholipid composition of this membrane. Changes in the ratio of phosphatidylcholine to phosphatidylethanolamine could affect ATP production by regulating electron transport chain activity, but the specific molecular mechanism remains unclear [56, 57]. Cardiolipin, a mitochondria-specific phospholipid, is essential for the assembly, stability, and functional integrity of respiratory chain supercomplexes, thereby supporting efficient OXPHOS [58–60]. LPCAT1 participates in the biosynthesis and fatty acid remodeling of cardiolipin [61]. Given our finding that LPCAT1 knockdown impairs OXPHOS, we speculate that LPCAT1 may influence the stability and activity of the ETC by modulating the composition and function of cardiolipin. Future in-depth lipidomic research into whether and how LPCAT1 shapes the mitochondria lipid microenvironment will help to more comprehensively elucidate its role in the progression of HNSCC.

Despite all the findings, the current study has some limitations. The hypothesis that LPCAT1 upregulates COX17 expression by promoting nuclear translocation of the transcription factor SP1 requires further mechanistic validation. Consistent with a previous study [36], our current data demonstrate a correlation between LPCAT1 expression levels and nuclear SP1 protein abundance; however, this remains indirect evidence. To establish a definitive regulatory mechanism, future studies should employ chromatin immunoprecipitation assays to confirm direct SP1 binding to the COX17 gene promoter. These experimental approaches will collectively provide robust functional evidence for this proposed regulatory axis.

Nevertheless, this study systematically demonstrates significant reprogramming of PC metabolism in HNSCC and identifies LPCAT1 as a key oncogenic driver. We demonstrate that LPCAT1 fuels tumor growth by upregulating COX17 expression, thereby enhancing mitochondrial complex IV activity and boosting oxidative phosphorylation efficiency. These results provide a mechanistic foundation for understanding metabolic vulnerabilities and suggest potential avenues for therapeutic intervention.

Based on these findings, we consider the LPCAT1-mediated metabolic pathway to hold clear translational potential. On one hand, targeting LPCAT1 or the downstream OXPHOS pathway may offer a novel therapeutic approach, particularly for HNSCC patients exhibiting a high oxidative phosphorylation metabolic phenotype. On the other hand, the dysregulated PC profiles identified and preliminarily validated in this study provide a potential source of metabolite biomarkers for non-invasive diagnosis of HNSCC. In the future, quantitative detection of specific PC molecules in serum or tissue samples using mass spectrometry in large clinical cohorts could enable the development of biomarker panels for early warning, prognosis assessment, or treatment response evaluation in HNSCC. Overall, further investigation and clinical translation of the LPCAT1–PC–OXPHOS axis may open new pathways toward improving the diagnosis and treatment of HNSCC.

## Materials and methods

### Tissue acquisition

The human HNSCC tissues used in this study were obtained from treatment-naïve patients who underwent surgical resection at the First Affiliated Hospital of Chongqing Medical University. Tumor tissues and matched adjacent normal mucosal samples were surgically resected from primary tumor sites and the corresponding normal mucosal regions, respectively. Individuals who had previously received neoadjuvant radiotherapy or chemotherapy were excluded. All tissues were immediately flash-frozen in liquid nitrogen post-resection and stored in liquid nitrogen for subsequent analyses. The collection and use of human tissue samples in this study were approved by the Institutional Review Board of the First Affiliated Hospital of Chongqing Medical University (approval No. 2021-433). All patients signed informed consent prior to surgery.

### Untargeted metabolomics with LC–MS/MS

Fifty normal tissues and eighty-one human HNSCC tissues (100 mg each) were sent to Applied Protein Technology (Shanghai, China) for LC–MS/MS-based untargeted metabolomics analysis according to the described protocol [62]. In brief, LC–MS/MS Analysis conducted using an UHPLC (1290 Infinity LC, Agilent Technologies) coupled to a quadrupole time-of-flight (AB Sciex TripleTOF 6600). Chromatographic separation was achieved on an ACQUITY UPLC BEH HILIC column (2.1 mm × 100 mm, 1.7 µm) with a mobile phase of (A) 25 mM ammonium acetate and 25 mM ammonium hydroxide in water and (B) acetonitrile. The gradient elution was as follows: 85% B for 1 min, linearly decreased to 65% B over 11 min, then reduced to 40% B and held for 4 min, and then increased to 85% in 0.1 min, followed by a 5-min re-equilibration. MS detection was performed in both ESI-positive and -negative modes with the source parameters set as: Gas1, 60; Gas2, 60; curtain gas, 30; source temperature, 600 °C; IonSpray Voltage, ±5500 V. MS-only spectra were acquired over the m/z range 60-1000 with an accumulation time of 0.20 s/spectrum. Auto MS/MS spectra were acquired over the m/z range 25-1000 with an accumulation time of 0.05 s/spectrum, using a collision energy of 35 ± 15 eV and a declustering potential of ±60 V.

### RNA sequencing and qPCR

Total RNA was extracted from fresh-frozen human HNSCC tissues or cells using the TRIzol® Reagent. RNA sequencing and bioinformatic analysis were performed by Majorbio (Shanghai, China). In brief, mRNA was enriched by poly(A) selection, fragmented, and reverse-transcribed into double-stranded cDNA using random hexamer primers. The cDNA underwent end-repair, A-tailing, and was size-selected (~300 bp), followed by PCR amplification. The final library was quantified and sequenced on an Illumina NovaSeq X Plus platform (2×150 bp). Differential expression analysis was conducted using DESeq2 (FDR < 0.05, |log2FC | ≥1). Kyoto Encyclopedia of Genes and Genomes (KEGG) pathway enrichment was analyzed via KOBAS (Bonferroni-corrected *P* value ≤ 0.05). Gene Set Variation Analysis (GSVA) was conducted on the Majorbio website (www.majorbio.com). For qPCR, 1 μg total RNA was reverse-transcribed into cDNA with RT Master Mix for qPCR II (HY-K0511, MCE), followed by qPCR using SYBR® Green qPCR Master Mix II (HY-K0523, MCE). Specific primer sequences are listed in Supplemental Table S1. GAPDH was used for normalization. Relative mRNA levels were calculated using 2^-ΔΔCt.

### Bioinformatics analysis

To characterize LPCAT1 expression profiles in HNSCC, transcriptomic data from the TCGA-HNSC cohort were analyzed using the GEPIA2 website (http://gepia2.cancer-pku.cn/). Protein-level evidence was obtained from the Human Protein Atlas database (https://www.proteinatlas.org/).

### Western blotting

Total protein was extracted from tissues and cells using RIPA lysis buffer. Protein samples were dissolved on an acrylamide gel and blotted wetly onto the PVDF membrane. After blocking, membranes were incubated with target-specific antibodies against LPCAT1 (1:2500, 16112-1-AP, Proteintech Group, IL, USA), COX17, (1:1000, 11464-1-AP, Proteintech Group), OXPHOS Cocktail (1:4000, PK30006, Proteintech Group) overnight at 4 °C. The next day, the membranes were probed with species-matched horseradish peroxidase-conjugated secondary antibodies. Immunoblots were visualized using chemiluminescent reagents. The intensity of bands was quantified using ImageJ (National Institutes of Health, Bethesda, MD).

### Immunohistochemistry staining

Paraffin-embedded tissue sections were deparaffinized and rehydrated in graded ethanol. Antigen retrieval was done in Tris-EDTA buffer (pH = 9). Endogenous peroxidase activity was blocked with 3% H₂O₂ at 37 °C for 15 minutes. Sections were incubated with primary antibodies against LPCAT1 (1:200, 16112-1-AP, Proteintech Group), Ki-67 (1:10,000, 27309-1-AP, Proteintech Group) at 4 °C overnight, followed by Polymer-HRP anti-mouse/rabbit secondary antibodies at 37 °C for 20 min. All sections were washed with phosphate buffer saline three times and visualized after staining with diaminobenzidine and hematoxylin.

### Immunofluorescence staining

Cells were initially fixed with 4% paraformaldehyde for 30 min, followed by permeabilization with pre-chilled methanol at −20 °C for 15 min. After blocking with 5% bovine serum albumin for 30 min, the cells were incubated overnight at 4 °C with primary antibodies against COX17 (1:100, 11464-1-AP, Proteintech Group), SP1 (1:200, ET1702-02, Huabio). Subsequently, the cells were incubated with fluorescent secondary antibodies for 60 min. Mitochondria were visualized with MitoTracker Green (Beyotime) for 20 min, and nuclei were stained with DAPI (Beyotime) for 10 min. All images were acquired using an Olympus laser scanning confocal microscope.

### Cell lines and cell culture

The FaDu and SCC15 HNSCC cell lines were purchased from the Cell Bank of the Chinese Academy of Sciences. All cells used in this study were tested for mycoplasma contamination and authenticated by STR sequencing when we purchased them. Cells were maintained in high-glucose Dulbecco’s Modified Eagle Medium (Gibco) supplemented with 10% fetal bovine serum (MCE). All cultures were kept at 37 °C in a humid environment with 5% CO_2_. For supplementation with exogenous DPPC, and cells were treated with 10 μM DPPC (HY-109506, MCE).

### Small interfering RNA (siRNA) and cell transfection

siRNA was synthesized by OBiO Technology and Sangon Biotech (Shanghai, China). siRNA sequences are provided in the Supplementary Table S2. siRNA transfection was performed in 24-well using Lipofectamine RNAiMAX (#13778030, Invitrogen) according to the manufacturer’s protocol. Complexes were formed by incubating 6 pmol siRNA with 1 μL Lipofectamine RNAiMAX in 100 μL Opti-MEM per well for 20 minutes at room temperature. Cells were then seeded directly onto the complexes at a density targeting 30–50% confluency. Cells were cultured for 24 h in serum-free medium. Twenty-four hours later, the transfection medium was replaced with fresh complete culture medium. At 48 h or 72 h after transfection, follow-up experiments were conducted.

### Lentivirus vector and cell infection

Lentiviruses were used to transduce short hairpin RNA (shRNA) and overexpression constructs. The viruses were constructed by GeneChem Company (Shanghai, China). The target sequences of LPCAT1 have been detailed in Table S2. Briefly, HNSCC cells were seeded in six-well plates (5 × 10⁴ cells/well) and infected at approximately 30% confluence. Lentiviral vectors for LPCAT1 knockdown (shRNA) or overexpression, along with HiTransG A enhancer, were applied at an MOI of 10. Following 16-h incubation, the viral medium was replaced with fresh culture medium. Stable cell lines were then selected with 2 μg/mL puromycin for 1 week.

### Cell viability

The cell viability was tested by CCK-8 assay (Dojindo, Japan). Cells were plated in 96-well plates (1000 cells/well) and allowed to adhere. CCK-8 solution (1:10 in culture medium) was added to replace the existing medium. Following a 1-h incubation at 37 °C, absorbance at 450 nm was recorded using a microplate reader.

Live and dead assay was performed using a Calcein/PI Cell Viability/Cytotoxicity Assay Kit (Beyotime). After transfection with siRNA in 96-well plates, 100 μL of Calcein AM/PI detection working solution was added. The plates were incubated at 37 °C in the dark for 30 min. Live and dead cells were subsequently visualized and quantified using a fluorescence microscope.

For the plate colony formation assay, Cells were seeded in 12-well plates (1000 cells/well) and incubated for 10 days. Changed the medium every 3 days. Colonies were fixed with 4% paraformaldehyde, stained with 0.1% crystal violet. Images of representative colonies were documented using an inverted phase-contrast microscope. The number of clones was detected by ImageJ software.

### Scratch wound assay

Cell migration capacity was assessed using scratch wound assays. Confluent monolayers of cells were scratched with a sterile 10-μL pipette tip. Cells were maintained in serum-free medium under standard culture conditions. Wound closure was quantified by imaging the denuded area at 0 h and 24 h post-scratch using microscopy. Migration rates were calculated using ImageJ software.

### Transwell invasion assay

Invasion capacity was assessed using Matrigel-coated Transwell chambers. Briefly, 100 μL Matrigel matrix (1:40 dilution in serum-free medium; Beyotime) was polymerized in the upper chamber. In total, 200 μL serum-free medium containing 5 × 10^4^ cells/well was added to the upper chamber, while the lower chamber contained 600 μL Dulbecco’s Modified Eagle Medium supplemented with 15% fetal bovine serum as a chemoattractant. After 24 h, non-invading cells in the upper chamber were removed mechanically. Invaded cells were fixed with 4% formaldehyde, stained with 0.1% crystal violet, and quantified by counting three randomly selected fields.

### Xenograft tumor model

Orthotopic tongue xenograft models were established in 5–6-week-old male BALB/c nude mice purchased from GemPharmaTech. Mice were bred under specific-pathogen-free conditions. Before the experiment, mice were randomized and grouped according to body weight (*n* = 5/group). Briefly, the mice were anaesthetized with tribromoethanol (DW3112, Dowobio, Shanghai, China). In total, 5 × 10⁵ FaDu-shNC or FaDu-shLPCAT1 cells suspended in 15 μL PBS were injected into the left lingual submucosa. Body weight was measured every 5 days. On day 15, a subset of mice exhibited a weight loss of more than 20% relative to baseline. On the same day, all mice received an intraperitoneal injection of 150 mg/kg D-luciferin (ST196, Beyotime), followed by bioluminescence imaging using the Aniview imaging system. Then, all mice were euthanized for tumor excision. All animal experiments were conducted according to the ethical policies and procedures approved by the Institutional Animal Care Committee of the Second Affiliated Hospital of Chongqing Medical University (approval No. IACUC-SAHCQMU-2025-0014).

### Mitochondrial membrane potential assay

The mitochondrial membrane potential was measured using the JC-1 Mitochondrial Membrane Potential Assay Kit (HY-15534, MCE). Briefly, cells were seeded at a concentration of 5 × 10^5^ cells/well in six-well plates. After the cells adhered to the wall, incubated with JC-1 staining solution for 10 min at 37 °C in the dark. The fluorescence was observed with fluorescence microscopy.

### Cytochrome c oxidase activity assay

Cytochrome c oxidase (Cco) activity was detected using a commercial kit (ADS-W-X010, Aidisheng Biologic Technology, Jiangsu, China) according to the manufacturer’s instructions. Briefly, 5 × 10^6^ cells were homogenized in ice-cold extraction buffer, followed by centrifugation to isolate mitochondria. The mitochondrial pellet was resuspended and then sonicated on ice. The reaction mixture was sequentially incubated with the corresponding reagents at room temperature. The absorbance at 550 nm was measured (A1), and then the absorbance was measured again after incubation at 37 °C for 15 min (A2). The activity was calculated as follows: Complex IV activity (nmol/ minute/10^4^ cells) = 0.025 × ΔA, where ΔA = A1 − A2. All steps were performed at 4 °C.

### ATP measurement

Cellular ATP was measured using a luminescence-based assay (S0026, Beyotime) according to instructions. Briefly, after cell lysis and centrifugation, the supernatant was collected. Then, 20 µL of the supernatant was mixed with 100 µL of working solution, mixed, and immediately subjected to measurement using a Spectramax (Molecular Devices) microplate reader to read the luminescence. The protein concentration in the samples was determined using the BCA method for normalization.

### Measurement of mitochondrial respiration

Mitochondrial stress testing was assessed using the Seahorse XFe Analyzer (Agilent Technologies) according to the manufacturer’s protocol. Cells were plated at a density of 40,000 cells per well in DMEM supplemented with 10% FBS and incubated overnight. On the day of the experiment, the cells were washed, and the medium was replaced with DMEM supplemented with 10 mM glucose, 2 mM glutamine, and 1 mM pyruvate. A standard mitochondrial stress test was performed using sequential injections of 1.5 μM oligomycin, 2 μM carbonyl cyanide-4-(trifluoromethoxy) phenylhydrazone (FCCP), and 0.5 µM for both rotenone and antimycin A. Monitor OCR parameters in real time. All OCR values were normalized to the total protein detected by the BCA method.

### Transcription factor prediction of COX17

Potential transcription factors regulating COX17 were predicted using three independent databases: GeneCards (https://www.genecards.org/), hTFtarget (https://guolab.wchscu.cn/hTFtarget/), and KnockTF (http://www.licpathway.net:8081/KnockTFv2/). Following the respective protocols of each database, we obtained candidate TF lists. Overlapping transcription factors of the three predicted TF sets were identified by a Venn diagram.

### Statistical analysis

Data are presented as mean ± SD or median with interquartile range. Statistical analyses were performed using GraphPad Prism (v9.3.1) with significance defined as *P* < 0.05. Normality was assessed with the Shapiro–Wilk test. Homogeneity of variances was tested using either the F-test or the Brown-Forsythe test. Comparisons between two groups utilized unpaired Student’s *t* tests or Mann–Whitney test. Multiple comparison testing was done by one-way ANOVA or two-way ANOVA. Orthogonal Partial Least Squares Discriminant Analysis (OPLS-DA) was performed in Simca (v14.1). The permutation test (200 permutations) was performed to validate the OPLS-DA model.

## Supplementary information

supplement material

Original Data

## Data Availability

RNA-sequencing data have been deposited to NCBI Sequence Read Archive repository under BioProject ID PRJNA1396908.
