# Supplementary material for: Lysophosphatidylcholine acyltransferase 1 promotes head and neck squamous cell carcinoma progression by enhancing COX17-dependent oxidative phosphorylation
Source: Cell Death Discov. 2026 Mar 6;12:139. doi: 10.1038/s41420-026-02994-3 (PMC13039686; doi:10.1038/s41420-026-02994-3)
Supplement: Supplementary file 1 — supplement material [file 41420_2026_2994_MOESM1_ESM.docx]

| gene | primer sequence | |
| --- | --- | --- |
| LPCAT1 | Forward | 5’-CTGACTTCCACAGGTTTGC-3’ |
|  | Reverse | 5’-TGGGTCCTAATCCAGCTTC-3’ |
| GAPDH | Forward | 5’-GGGGCTCTCCAGAACATC-3’ |
|  | Reverse | 5’-TGACACGTTGGCAGTGG-3’ |
| COX17 | Forward | 5’-TGGCATAGATTTGGCTGTC-3’ |
|  | Reverse | 5’-CAGACCCGGCATCTTTC-3’ |
| NDUFA8 | Forward | 5’-GTTGGGCAGGGCAATAC-3’ |
|  | Reverse | 5’-GGGCAGCTCCACTATCC-3’ |
| NDUFB7 | Forward | 5’-GGCACGACTGGGACTACTG-3’ |
|  | Reverse | 5’-CAACTCTGCCGCCTTCTT-3’ |
| UQCRQ | Forward | 5’-GGATCGGTGACTGTGGAG-3’ |
|  | Reverse | 5’-AGTGAAGACGTGCGGATAG-3’ |
| NDUFA2 | Forward | 5’-TTTTCCCTCCGACCAAC-3’ |
|  | Reverse | 5’-CCCTAAAGCCTAGCCCAT-3’ |
| COX5B | Forward | 5’-CTGGGTTGGAGAGGGAGA-3’ |
|  | Reverse | 5’-GTTGGAGATGGAGGGGACT-3’ |
| NUDFA1 | Forward | 5’-ATGTGGTTCGAGATTCTCCCC-3’ |
|  | Reverse | 5’-CCTGTGGATGTACGCAGTAGC-3’ |
| UQCR11 | Forward | 5’-GCCACAGTTCCTTCCCA-3’ |
|  | Reverse | 5’-GCTTGCTCCTTCCTCTCC-3’ |
| MRPS12 | Forward | 5’-TGGCCTCAACACGTCCCTAA-3’ |
|  | Reverse | 5’-TCTTCGGCTTGCGGGTAAAC-3’ |
| COX7A2 | Forward | 5’-CTCGGAGGTAGTTCCGGTTC-3’ |
|  | Reverse | 5’-TCTGCCCAATCTGACGAAGAG-3’ |
|  |  |  |

Supplementary table 1 Primer sequences for qPCR

| siRNA/shRNA | sequence | |
| --- | --- | --- |
| LPCAT1-siRNA/shRNA | sense | 5’-UACCCGGAUCAGACACAUUUCTT-3’ |
|  | anti-sense | 5’-GAAAUGUGUCUGAUCCGGGUATT-3’ |
| COX17-siRNA | sense | 5’-GGAAUGCAUGAGAGCCCUA-3’ |
|  | anti-sense | 5’-UAGGGCUCUCAUGCAUUCC-3’ |
| Negative control | sense | 5’-UUCUCCGAACGUGUCACGUTT-3’ |
|  | anti-sense | 5’-ACGUGACACGUUCGGAGAATT-3’ |

Supplementary table 2 Sequences for LPCAT1 and COX17 siRNA/shRNA


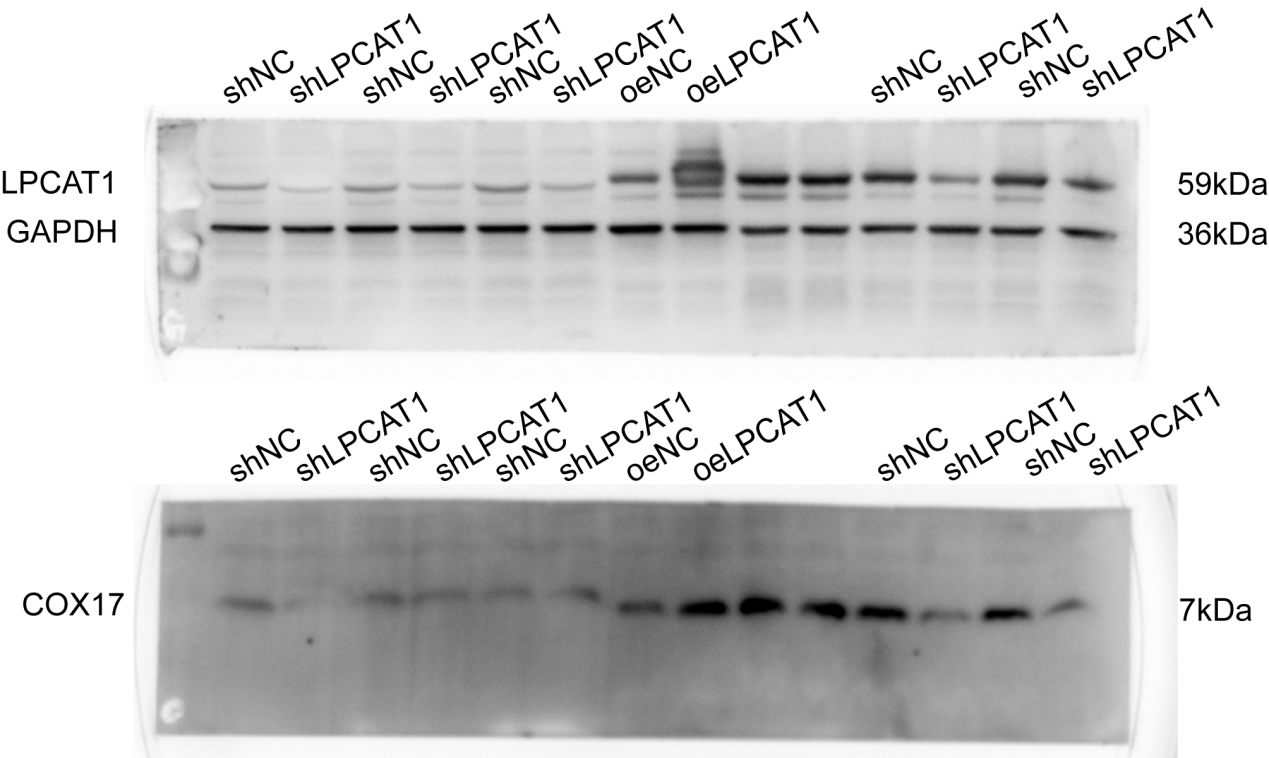


Supplementary Figure s1 COX17 protein were measured by western blotting after LPCAT1 knockdown or overexpression.


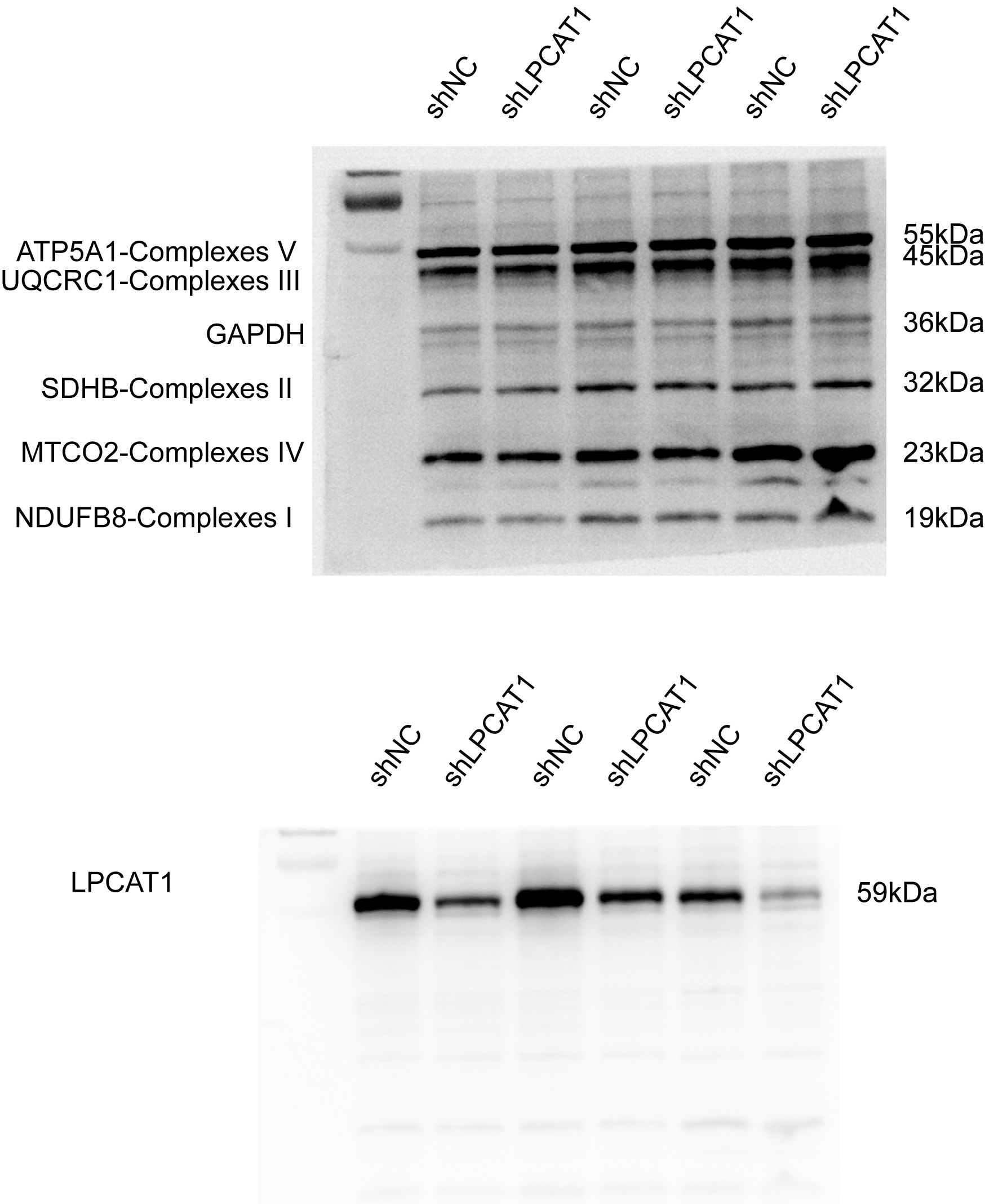


Supplementary Figure s2 Western blotting analysis of electron transport chain complex core subunits (I-V)
