# Supplementary material for: Lysophosphatidylcholine acyltransferase 1 promotes head and neck squamous cell carcinoma progression by enhancing COX17-dependent oxidative phosphorylation
Source: Cell Death Discov. 2026 Mar 6;12:139. doi: 10.1038/s41420-026-02994-3 (PMC13039686; doi:10.1038/s41420-026-02994-3)

COX17 protein were measured by western blotting after LPCAT1 knockdown or overexpression.


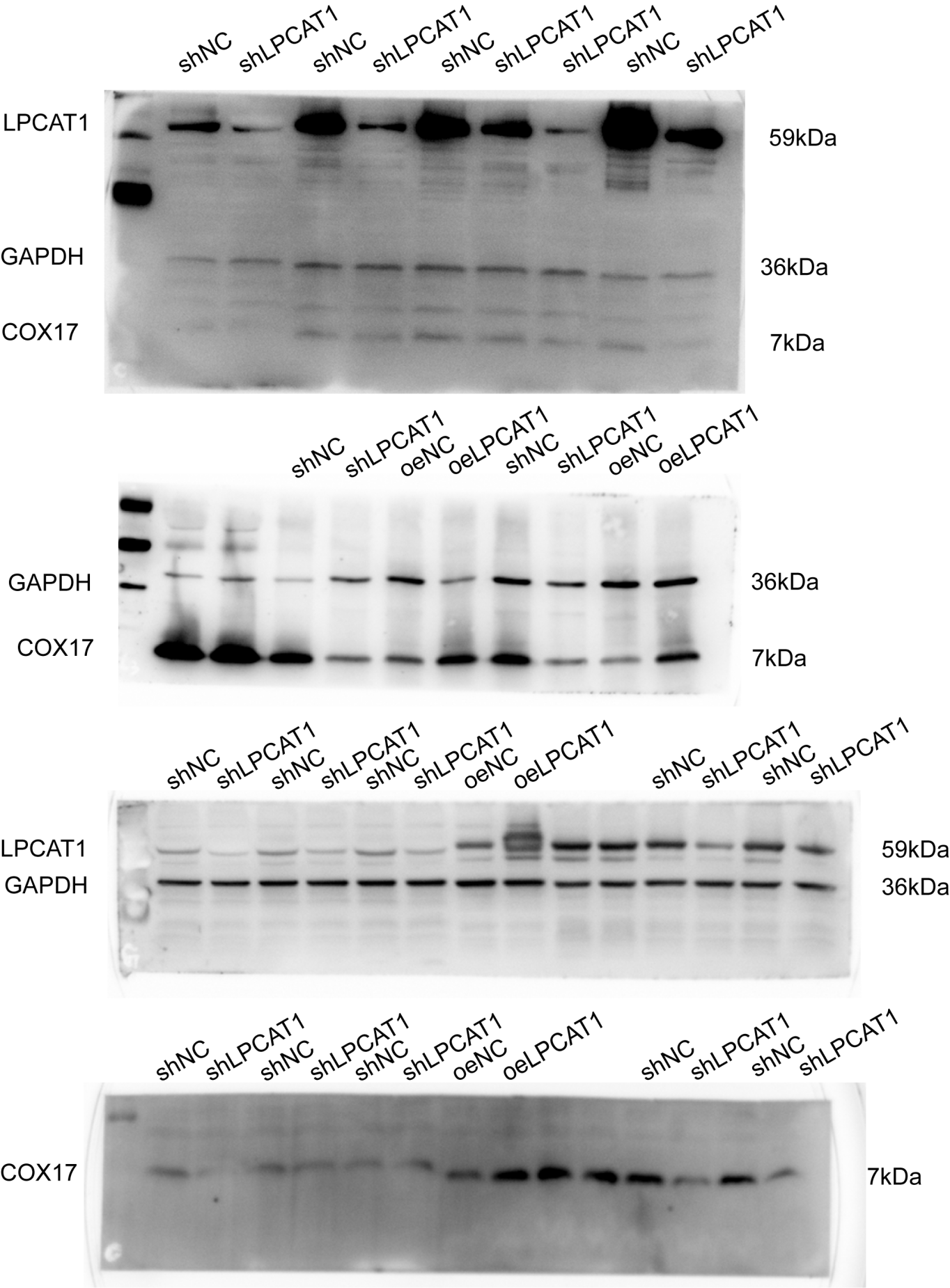


Western blotting analysis of electron transport chain complex core subunits (I-V)


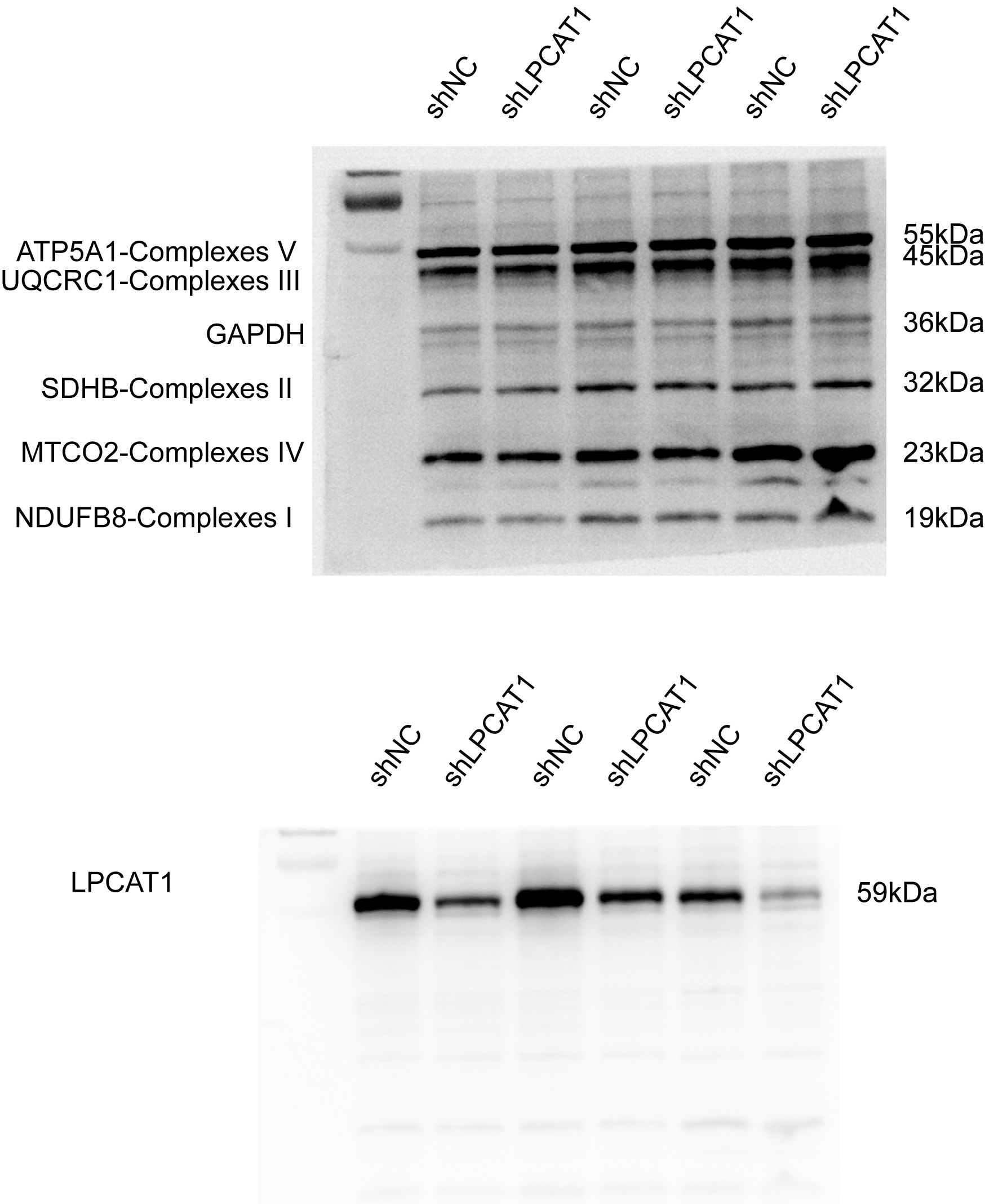

Supplement: Supplementary file 2 — Original Data [file 41420_2026_2994_MOESM2_ESM.docx]
